# Multiparametric MRI-based differentiation of WHO grade II/III glioma and WHO grade IV glioblastoma

**DOI:** 10.1038/srep35142

**Published:** 2016-10-14

**Authors:** Benedikt Wiestler, Anne Kluge, Mathias Lukas, Jens Gempt, Florian Ringel, Jürgen Schlegel, Bernhard Meyer, Claus Zimmer, Stefan Förster, Thomas Pyka, Christine Preibisch

**Affiliations:** 1 Department of Neuroradiology, Klinikum rechts der Isar, TU München, Germany; 2 Department of Nuclear Medicine, Klinikum rechts der Isar, TU München, Germany; 3 Department of Neurosurgery, Klinikum rechts der Isar, TU München, Germany; 4 Department of Neuropathology, Klinikum rechts der Isar, TU München, Germany; 5 TUM Neuroimaging Center (TUM-NIC), Klinikum rechts der Isar, TU München, Germany

## Abstract

Non-invasive, imaging-based examination of glioma biology has received increasing attention in the past couple of years. To this end, the development and refinement of novel MRI techniques, reflecting underlying oncogenic processes such as hypoxia or angiogenesis, has greatly benefitted this research area. We have recently established a novel BOLD (blood oxygenation level dependent) based MRI method for the measurement of relative oxygen extraction fraction (rOEF) in glioma patients. In a set of 37 patients with newly diagnosed glioma, we assessed the performance of a machine learning model based on multiple MRI modalities including rOEF and perfusion imaging to predict WHO grade. An oblique random forest machine learning classifier using the entire feature vector as input yielded a five-fold cross-validated area under the curve of 0.944, with 34/37 patients correctly classified (accuracy 91.8%). The most important features in this classifier as per bootstrapped feature importance scores consisted of standard deviation of T1-weighted contrast enhanced signal, maximum rOEF value and cerebral blood volume (CBV) standard deviation. This study suggests that multimodal MRI information reflects underlying tumor biology, which is non-invasively detectable through integrative data analysis, and thus highlights the potential of such integrative approaches in the field of *radiogenomics*.

How glioma histology and genotype is reflected in imaging has been subject of an increasing number of recent studies^1^. Such information may on the one hand support initial clinical decision making and on the other hand potentially allow non-invasive follow-up of changes of tumor biology over time, for example during therapy. With the advent of novel therapeutic strategies specifically targeting defined genomic lesions^2^, the interest in this field has grown even further.

Traditionally, anatomic imaging sequences such as T1 and T2 weighted (T1w, T2w) images have been used to differentiate high-grade from low-grade gliomas^3^ or to identify defined genomic aberrations such as combined 1p/19q loss in oligodendroglial tumors^4^. However, significant overlap for imaging characteristics between WHO grades and genotypes (such as the presence of contrast enhancement in low-grade gliomas) limits the univariate use of T1w and T2w sequences as a definite predictor of grade in clinical practice^5^,^6^,^7^. Pivotal to the field of *radiogenomics* has, therefore, been the development and refinement of physiological MRI techniques visualizing key oncogenic processes such as invasion, angiogenesis or hypoxia. Of these, MRI based measures of tumor perfusion, including both dynamic susceptibility contrast (DSC) and dynamic contrast enhancement (DCE) perfusion, have probably been studied most extensively. Quite consistently, increased perfusion metrics have been associated with shortened progression-free^8^ and overall^9^,^10^ survival in newly-diagnosed glioma. Furthermore, several authors have suggested association of perfusion metrics and molecular biomarkers in glioma, such as epidermal growth factor receptor (EGFR) status^8^ or WHO grade (III vs. IV)^9^.

We have recently established a novel BOLD (blood oxygenation level dependent) based MRI method for the measurement of vascular deoxygenation, i.e. relative oxygen extraction fraction (rOEF), in glioma patients. In a subgroup of patients, this non-invasive measure of hypoxia also correlated reasonably well with hypoxia-inducible factor 1α (HIF1α) immunohistochemistry stainings^11^. Hypoxia has long been known to play a central role in gliomagenesis and is related to a more malignant tumor phenotype and therapy resistance^12^. Accordingly, several studies have shown that HIF1α protein expression increases with WHO grade^13^.

In order to evaluate the large feature vectors resulting from integrative analysis of image features and their association with underlying biology, machine-learning algorithms such as random forests and support vector machines clearly outperform traditional approaches such as correlation analysis and are increasingly being used in fields such as genomics^14^ and imaging analysis^15^.

We hypothesized that integrative analysis of multimodal MRI data, considering anatomic sequences (T1 and T2 weighted) as well as physiological imaging (DSC perfusion and hypoxia imaging), reflects the underlying glioma biology and hence allows for its non-invasive detection.

## Results

### Patient characteristics

In total, this study included 37 patients (24 male, 13 female; mean age 58 years), 27 of which had a glioblastoma (73%; WHO grade IV), five an anaplastic glioma (13.5%; WHO grade III) and five a diffuse glioma (13.5%; WHO grade II). Of the grade II and III glioma, seven were diagnosed with an astrocytoma, two with a mixed oligoastrocytoma and one patient with a pure oligodendroglioma. *Isocitrate dehydrogenase* (*IDH*) mutation status was available for 35/37 patients: 31 patients were *IDH* wild type, while four patients (two WHO grade II glioma, two WHO grade III glioma) carried a mutant *IDH* allele.

### Multimodal image feature analysis

**Figure 1:**
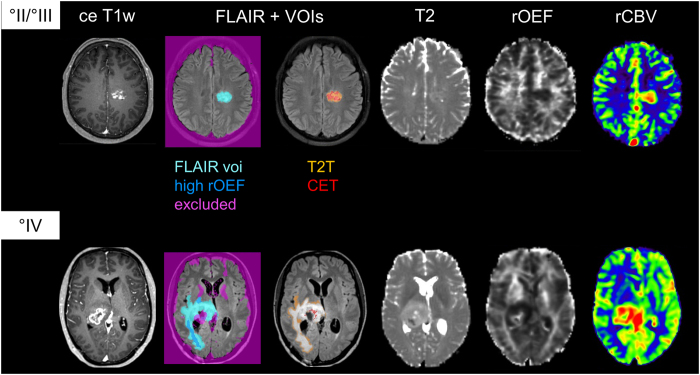
Examples of a WHO grade II/III glioma (top row) and WHO grade IV glioblastoma (bottom row) and VOI definition. Sequences shown are contrast-enhanced T1w, FLAIR, T2, rOEF and CBV.

We performed multimodal MRI on all 37 patients, including rOEF as a marker for hypoxia and perfusion imaging (Fig. 1). For image analysis, volumes of interest (VOI) were manually defined by CP and summary and volume statistics were extracted for these VOIs in all sequences, resulting in a total of 116 features (see methods for details).

Based on recent work on the (epi)genetic basis of gliomas^16^,^17^,^18^ which indicate that WHO grade II and III gliomas can be rather subdivided by molecular status than WHO grade, we grouped these tumors as opposed to WHO grade IV glioblastoma.

**Figure 2:**
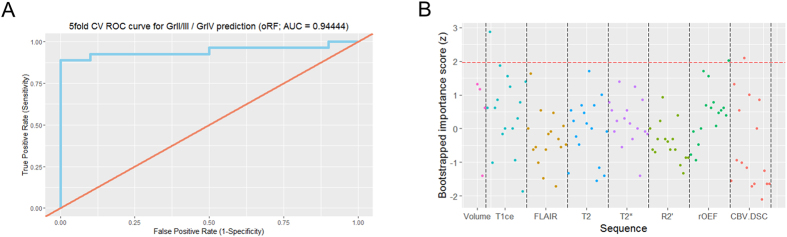
(**A**) Five-fold cross-validated receiver operating characteristic (ROC) curve for the random forest classifier predicting WHO grade. (**B**) Plot of the z-transformed bootstrapped mean feature importance scores. Here, each dot represents a feature, with the feature importance plotted on the y axis.

**Figure 3:**
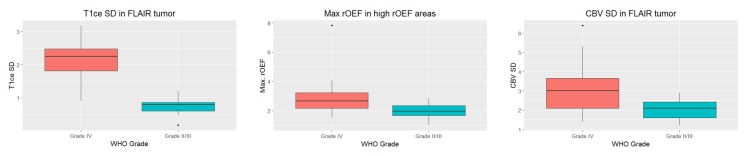
Box plots of the three most important features for the differentiation between grade II/III glioma and grade IV glioblastoma.

We trained an oblique random forest with 300 trees, using logistic regression as the node model. To account for the bias in prediction accuracy, we performed a five-fold cross-validation to estimate classifier performance. The resulting model had an area under the curve of 0.944, with 34 of 37 patients correctly classified (Fig. 2A; sensitivity 0.8889, specificity 1, positive predictive value 1, negative predictive value 0.7692). In this analysis, three patients with a glioblastoma got misclassified as grade II/III glioma. Of these, all three were *IDH* wild type and also otherwise showed no peculiarities. To investigate the features most important for this classification, we calculated the mean importance score from 1000 bootstrap iterations, using the same model parameters as above. Figure 2B displays the z-transformed mean importance scores for all 116 features. Of these, three features had a z-transformed importance score >1.96: Standard deviation of the contrast-enhanced (ce) T1w signal and rCBV in FLAIR-hyperintense tumor as well as maximum rOEF signal in the high rOEF VOI, which generally broadly overlapped with edema (Fig. 3). For all these features, values were higher in glioblastoma compared to WHO grade II/III glioma. Table 1 summarizes these features.

In our cohort, only four of 37 patients carried a mutant *IDH* allele. This low frequency prohibited training a prediction model for *IDH* mutational status in our cohort.

## Discussion

Novel MR imaging sequences reflecting underlying oncogenic processes have greatly advanced the field of *radiogenomics*. Here, we explored the ability of a machine-learning classifier based on an extensive multimodal MR imaging data set to distinguish between WHO grade II/III glioma and WHO grade IV glioblastoma.

For this study, we grouped WHO grade II and III gliomas as opposed to WHO grade IV glioblastomas. In the current fourth edition of the WHO classification^19^, signs of anaplasia and mitotic activity distinguish WHO grade II and III glioma. However, both criteria are rather subjective and prone to relevant inter-observer variability^20^,^21^, while the diagnosis of WHO grade IV glioblastoma, requiring the presence of necrosis and neoangiogenesis, is more reliable. Furthermore, recent advances in our understanding of the complex (epi)genetic basis of gliomas have led to the identification of several molecular subtypes associated with biology and prognosis^16^,^17^,^18^, and have shown that outcome differences between WHO grade II and III glioma more rely on the distribution of these molecular subgroups than on biological differences between WHO grade II and III glioma per se^22^, suggesting that grade II and III glioma may in fact be a single entity subdivided by molecular parameters. Glioblastoma, on the other hand, still have a worse prognosis than WHO grade II and III glioma, even when accounting for molecular parameters^17^. Based on these considerations and in accordance with large cohorts such as the cancer genome archive (TCGA), which also group WHO grade II and III glioma as “lower grade glioma” as opposed to grade IV glioblastoma, we also grouped our samples accordingly.

We identified three imaging features most important for the differentiation between WHO grades: One feature from a conventional anatomic sequence (ce T1w) and two features from physiological imaging (rCBV, rOEF). Using only image features derived from anatomic sequences to distinguish gliomas of different WHO grades is known to be limited by significant overlap between WHO grades^3^,^6^,^7^. Therefore, the use of several physiological imaging sequences, better capturing underlying tumor biology, has been explored. Of these, perfusion imaging has maybe been studied most extensively for its use in MRI-based glioma grading^23^,^24^,^25^. Most authors found maximum CBV values to be a significant discriminator between tumor grades (with higher values in higher WHO grades), while in our cohort, the standard deviation of the CBV was more important. This may be related to a finding of an earlier study, where we found maximum CBV to be reflective of *IDH* status^26^, and in our cohort, only four patients carried an *IDH* mutation. We hypothesize that standard deviation of the CBV in the FLAIR-hyperintense tumor reflects intratumoral heterogeneity, which is known to be a prominent feature of glioblastoma, both histopathologically and molecularly^27^. Maximum relative oxygen extraction fraction (rOEF) was higher in glioblastoma compared to WHO grade II/III glioma (Fig. 3, Table 1). Considering the preliminary correlation between HIF1α expression (as per immunohistochemistry) and areas of high rOEF^11^, this feature might well reflect hypoxic areas, again highly characteristic of glioblastoma and thus aid in the differentiation between WHO grade II/III glioma and WHO grade IV glioblastoma. Interestingly, high rOEF values were most prevalent in peritumoral edema, which also fits with recent observations of Jensen *et al*. who demonstrated expression of hypoxia markers (HIF1α and VEGF) in peritumoral edema^28^.

Only four of the ten WHO grade II and III patients in our cohort carried a mutant *IDH* allele, while large-scale population-bases studies suggest that between 60–80% of these tumors should be *IDH* mutant^29^. Unfortunately, this low number of *IDH* mutations also precluded training a classifier for the detection of *IDH* mutation status. A possible explanation for the lower number of *IDH* mutant patients in our cohort might lie in the selection criteria for this study. In this cohort, we aimed to include mostly patients with a suspected high grade glioma, because we wanted to correlate hypoxia as detected by rOEF with a PET-based measure of hypoxia, [^18^F]-FMISO.

Unraveling such meaningful correlations in complex multivariate features sets requires elaborate computational algorithms that go beyond standard univariate statistical tests. The random forest algorithm excels in feature extraction and classification tasks for data sets with few observations but high-dimensional feature vectors – a property of most data sets used in *radiogenomics* studies, as well as ours. Here, we used a random forest implementation with “oblique” node models^30^ that overcomes deficiencies of the regular random forest when dealing with correlated features, which also is a typical property of an imaging data set in general and ours in particular. Importantly, such algorithms allow to input a high-dimensional data set without prior selection of “candidate sequences”, by automatically weighing each feature, and hence leverage the full information contained in the data set.

Five-fold cross-validation yielded an 91.8% accuracy (34 of 37 patients correctly classified), suggesting that advanced MR imaging indeed is able to predict underlying biology. However, the lack of an independent validation cohort as well as the relatively small sample size are limitations of our work and caution against strong conclusions, though we tried to account for this by bootstrapping and cross-validating all steps in training and evaluating our classifier. Furthermore, this study is retrospective in nature and hence suffers from limitations associated with retrospective data sets.

In summary, multimodal MR imaging analysis using machine-learning techniques holds great promise for advancing the field of *radiogenomics*, enabling non-invasive insight into tumor biology, and possibly in the future informing clinical decision making. Our results warrant future studies.

## Methods

### Subjects

47 patients with suspected glioma were examined using an extended MRI protocol. Data of ten patients had to be excluded because of other or lacking diagnosis (n = 4), severe motion artifacts in R2′ measurement (n = 2) and technical problems or motion in the DSC-based CBV measurement (n = 4). All patients provided informed written consent. The study was approved by the ethics committee of the TU Munich and carried out in accordance with the approved guidelines.

### MRI study protocol

MRI examinations were performed on a clinical 3 T mMR Biograph scanner (Siemens Medical Solutions, Germany). The advanced clinical MRI protocol comprised R2′ mapping (voxel size 2 × 2 × 3 mm^3^, matrix 128 × 128, 30 slices) by separate acquisition of a multi-gradient echo (12 echoes, TE1 = 5 ms, ΔTE = 5 ms, TR = 1950 ms, α = 30°, exponential excitation pulse, rapid flyback, acq. time 4:08 min) and a multi-echo TSE sequence (8 echoes, TE1 = 16 ms, ΔTE = 16 ms, TR = 4040 ms, acq. time 5:04 min). Relative cerebral blood volume (rCBV) was obtained by dynamic susceptibility contrast (DSC) imaging (single-shot GE EPI: TR = 1500 ms, TE = 30 ms, α = 90°, 60–80 dynamics) during a bolus injection of 15 ml Gd-DTPA (prebolus of 7.5 ml)^31^. Calculation of MRI parameter maps was performed using custom programs in Matlab (MathWorks, Natick, Massachusetts, USA) as described previously^11^,^32^. In short, SPM8 (www.fil.ion.ucl.ac.uk/spm, using default parameters) was employed for spatial coregistration (rigid, 6 degrees of freedom) of the different image modalities (prior to *rOEF* calculation and VOI definition) and realignment (motion correction of DSC-MRI time courses if necessary). rCBV parameter maps were obtained by numerical integration of ΔR2*(t)^31^ and normalized by assuming a value of 1.5% for healthy white matter^32^. T2* evaluation included correction for motion^33^ and magnetic background gradients^34^ and T2 fitting was restricted to even echoes^11^,^32^. *rOEF* =* R2′/(c· rCBV)* was calculated from *R2* = (1/T2*) − (1/T2) and rCBV using *c* =* 4/3·π·γ·∆χ·B*_*0*_ = 317 Hz at 3T^11^,^32^ (with susceptibility difference between oxygenated and deoxygenated blood *∆χ* and gyromagnetic ratio *γ*). T1, T2, FLAIR and T2* images were normalized by computing a standard score 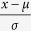 for tumor VOIs, using mean and standard deviation from healthy, non-tumorous brain. Using thresholding, manual editing, and logical VOI operations (difference and intersection), volumes of interest (VOI) were defined with Vinci (http://www.nf.mpg.de/vinci3) as described previously^11^. Briefly, a comprehensive FLAIR-hyperintense tumor VOI (FLAIR) was defined first, comprising tissue with any tumor related changes. Next, VOIs of solid appearing T2 visible tumor (T2T) and areas of contrast enhancement (in the T1w sequence (contrast enhancing tumor, CET) were defined to be mutually exclusive inside this FLAIR VOI. Furthermore, areas with high rOEF values (rOEF greater than mean + one standard deviation of healthy tissue) were defined (see Fig. 1 for illustration). Special care was taken to exclude artifacts (areas with necrosis, bleeding, iron deposition, macroscopic susceptibility perturbation). Within Vinci the VOI statistics functionality was then used to apply these VOIs to the spatially coregistered MR images and parameter maps, and extract the respective mean, standard deviation, minimum and maximum values together with the VOI volume for each sequence (contrast-enhanced T1w, FLAIR, T2, T2*, R2′, rOEF and CBV) and VOI, resulting in a total of 116 features.

### Statistical analysis

For supervised analysis of this high-dimensional data set, we trained an oblique random forest classifier^30^ with 300 trees (https://cran.r-project.org/package=obliqueRF). To validate this model, we performed a five-fold cross-validation; only cross-validated performance measures are reported, using the *pROC* and caret packages (https://cran.r-project.org/package=pROC & https://cran.r-project.org/package=caret)^35^. Using a bootstrapping approach (with 1,000 iterations), we calculated feature importance scores to identify the features most important for classification from the oblique random forest. Briefly, importance counts how often a variable was deemed relevant (at 0.05 level) when chosen for a split at a node (increasing the importance value by 1) and how often it was irrelevant for the split (decreasing by 1). For the most important features (bootstrapped standardized z score >1.96), effect size (Cohen’s d) was calculated and Welch’s t test performed. All tests were two-sided and carried out using R version 3.2^36^.

### Data availability

Phenotype data and anonymized & skull-stripped MR images are available upon request at figshare (https://figshare.com/articles/MRI_data_for_SciRep/3980175).

## Additional Information

**How to cite this article**: Wiestler, B. *et al*. Multiparametric MRI-based differentiation of WHO grade II/III glioma and WHO grade IV glioblastoma. *Sci. Rep.* **6**, 35142; doi: 10.1038/srep35142 (2016).

## Figures and Tables

**Table 1:** Overview of the most important features.

Feature	Importance Score (z)	Cohen’s d	p value
Standard deviation of T1ce values in FLAIR-hyperintense tumor	2.877775	2.798253	<0.0001
Maximum rOEF value in areas of high rOEF signal	2.022619	0.839689	0.003764
Standard deviation of CBV in FLAIR-hyperintense tumor	2.100360	0.9144929	0.001831

ce, contrast-enhanced.
